# Traits explain sorting of C_4_ grasses along a global precipitation gradient

**DOI:** 10.1002/ece3.7223

**Published:** 2021-02-03

**Authors:** Emma C. Jardine, Gavin H. Thomas, Colin P. Osborne

**Affiliations:** ^1^ Department of Animal and Plant Sciences University of Sheffield Sheffield UK

**Keywords:** drought, grasses, precipitation niche, senescence, strategies, survival, traits

## Abstract

1. Species distributions are closely associated with moisture availability, but the underlying mechanisms remain unresolved. Drought relations are especially important for plants such as C_4_ grasses that dominate seasonally dry ecosystems. Here, we test the hypothesis that C_4_ grass species sampled across global precipitation gradients show variation in survival under drought that can be explained by their traits.
2. Our experiment subjected 18 C_4_ grass species to a lethal drought under controlled environmental conditions. The number of days until death was measured, along with root traits, senescence, and aspects of hydraulic function.
3. We identified two strategies: Drought‐avoiding species that stayed green as the water potential declined and drought‐tolerating species that senesced more quickly but could extend survival via drought‐tolerant meristems.
4. Plants that stay‐green for longer occupied drier habitats and had the longest survival under drought, facilitated by narrow root diameter and isohydric stomatal behavior. Plants that senesced quickly had thicker roots, an anisohydric strategy, and occupied wetter habitats.
5. Global distributions of C_4_ grasses can be predicted by variation in rates of senescence, meristem survival, root traits, and stomatal strategy, showing the value of these traits for understanding plant distributions in relation to climate.

## Graphical abstract

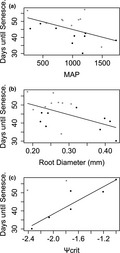

## 1 INTRODUCTION

Explaining why species occupy different environments is a central goal of ecology and understanding how plant traits mediate plant–climate relationships provides a way of answering this question. At a global scale, primary productivity and species distributions are correlated with gradients of precipitation (Currie & Paquin, 1987; Leith, 1975). These patterns are thought to be mediated by trade‐offs in species functional traits (Reich, 2014; Woodward, 1987). In habitats where water is not a limiting resource, competition excludes species and the ability to rapidly acquire resources and grow quickly is expected in the dominant species (Craine, 2009; Grime, 1977). However, species are known to separate along hydrological gradients (Silvertown et al., 2015) and soil water deficits can exclude plants that are not adapted to drought (Brenes‐Arguedas et al., 2009). Significant variation in drought performance has been observed among species, with those found in drier environments surviving longer under conditions of drought than species adapted to mesic conditions (Engelbrecht et al., 2005; Sack, 2004). This suggests that not only productivity under well‐watered conditions but also survival during drought events are important factors in determining species distributions along precipitation gradients.

Photosynthetic traits are closely associated with both growth and survival (Poorter & Bongers, 2006), and there is strong co‐ordination between photosynthetic characteristics and hydraulic function (Brodribb et al., 2002). When stomata are open for photosynthetic gas exchange, water loss through transpiration is inevitable, making stomatal conductance and hydraulic function inherently linked (Brodribb & Jordan, 2008). Stomatal regulation has therefore been proposed as the primary mechanism mediating plant mortality under drought (McDowell et al., 2008). A continuum between opposing hydraulic strategies has been described, with isohydric species at one end, and anisohydric species at the other. As soil water potential decreases under conditions of drought, isohydric species maintain midday water potential regardless of drought conditions by rapidly closing their stomata (McDowell et al., 2008). This strategy avoids hydraulic failure caused by cavitation (air entering the xylem vessels). The cost of stomatal closure is diminished carbon intake and, if a drought lasts longer than carbohydrate reserves, then carbon starvation may occur (Katul et al., 2003). Anisohydric species, by contrast, allow midday water potential to decline as available soil water declines, allowing continued photosynthetic carbon assimilation. However, under intense and prolonged drought, anisohydric species may suffer hydraulic failure caused by cavitation. A trade‐off has been described between hydraulic safety and efficiency (Skelton et al., 2015) which may provide adaptive benefits in areas of differing precipitation regime, depending on the length and predictability of drought events.

The link between mortality and hydraulic performance in different environments has largely been established for trees and relies upon the premise that hydraulic failure causes death. However, many species of grass, forbs, and shrubs have meristems (tissues capable of regrowth) either below ground or just above the soil which can produce new shoots after loss of above‐ground parts (Overbeck & Pfadenhauer, 2007). In such cases, the rates of stomatal closure may be decoupled from plant survival under conditions of declining soil moisture. A number of other mechanisms may also decouple or modify the stomatal behavior–mortality relationship. For example, leaf rolling allows gas exchange to continue, while reducing water loss (Kadioglu & Terzi, 2007; Knapp, 1985). Canopy senescence can be induced by drought stress and plays a major role in survival by remobilizing nutrients that have been accumulated in leaves. When this is accompanied by leaf shedding, water losses through transpiration are also avoided (Munne‐Bosch & Alegre, 2004). Leaf shedding is commonly found among species of mediterranean‐type and seasonal subtropical environments, and both deciduousness and a thickened taproot strongly predict survival along a precipitation gradient in tropical trees (Ackerly, 2004; Poorter & Markesteijn, 2008).

Plant roots play a role in plant growth via the uptake of water (and nutrients) and also tolerance to drought. Intraspecific dehydration tolerance has been associated with a conservative strategy of resource acquisition (low SLA and specific root length (Balachowski & Volaire, 2018). An intraspecific trade‐off between dehydration avoidance and dehydration tolerance has been observed, with thinner, denser roots associated with greater survival under drought and dehydration avoidance (Bristiel et al., 2019). A trend toward roots of finer diameter and higher SRL has been observed to correlate from tropical to temperate areas which are thought to be driven by climatic factors. Climatic factors have been suggested to have an important effect on root traits, with a trend toward finer diameter and higher SRL from tropical to temperate areas (Chen et al., 2013). However, far less is known about how root traits may vary between species along climatic gradients than is known for above‐ground traits (Laliberté, 2017).

Grasslands, in which the ground cover is dominated by C_4_ grass species, cover 20% of the vegetated land surface in regions where seasonal droughts occur. These regions also occupy a very broad precipitation range from ~200 mm MAP to ∼3,000 mm MAP (Scholes & Archer, 1997). These climate relationships make them an excellent system for studying large‐scale patterns of drought performance. Climate has long been considered a major factor in determining the global distributions of grass species (Hartley, 1952; Taub, 2000), and different phylogenetic clades have associations with areas of contrasting aridity at both global and regional scales (Edwards & Smith, 2010; Visser et al., 2012). Trait interactions with moisture availability may be a key factor in determining habitat associations of grasses. However, we currently have limited knowledge of the adaptive functional, physiological, and morphological traits underpinning these large‐scale patterns. This study aims to understand the traits underlying species differences in mortality during drought and to link these with the patterns of precipitation influencing spatial distributions of C_4_ savanna grasses. We hypothesize that mortality of C_4_ grasses under drought correlates with species distribution patterns. We measure time until senescence and death, stomatal conductance, predawn and midday water potential, leaf rolling, root diameter, and specific root length (SRL). We expect that species from arid areas will adopt an isohydric strategy, maintaining shoot water potential and green leaves, surviving longer under drought via early stomatal closure, and being characterized by low specific root length and narrow root diameter. Conversely, we expect plants from mesic habitats to adopt an anisohydric strategy, having a high specific root length and wide root diameter.

## 2 MATERIALS AND METHODS

### 2.1 Species selection and environmental variables

We designed a controlled environment experiment to test for differences in survival under drought and measured traits that might explain variation in survival. We sampled a total of 18 C_4_ grass species, chosen to include representatives from six independently derived lineages containing C_4_ species (Appendix 1), and also species whose distributions represent a wide gradient of MAP (mean annual precipitation). All of the species selected for study were determined as locally dominant based upon information from vegetation maps (e.g., White, 1983).

The realized precipitation niche of each species was quantified by mapping occurrence data from GBIF onto the climatic data from WorldClim (Hijmans et al., 2005). Point occurrence data from GBIF were cleaned so that the analysis only included records where the co‐ordinate reference was recorded to two decimal places or more, and where the GBIF country code matched the country of the co‐ordinate reference. The remaining numbers of geo‐referenced records for each species are listed in Appendix 1. Habitat data from floras were cross‐checked for each species to ensure plants from shallow, eroded soils or wetlands were excluded, as precipitation data would not accurately reflect the water available in the habitats of these species.

### 2.2 Drought experiment

Seeds were germinated in a controlled environment (MLR 352H, Sanyo, Osaka, Japan), on moist filter paper on petri dishes with a 16‐hr day length, a day/night temperature of 25/20°C, and 60% humidity. Hard‐coated seeds with a low germination rate were soaked in water that had boiled, then been left to cool for 10 min, followed by being soaked in cold water for 24 hr before being put into the chamber. Seedlings were transplanted once they had one fully expanded leaf into 1‐liter pots (length, 5 cm; width, 5 cm; and height, 40 cm) containing John Innes No.1 compost and 2.5 g of slow‐release fertiliser granules (Miracle Gro, Scotts, Marysville, Ohio, USA). The experiment took place in a controlled environment chamber (MTPS 120, Conviron, Winnipeg, Manitoba, Canada). A randomized block design was used to ensure there was no bias in the experiment due to environmental heterogeneity.

After transplanting, plants were grown on in the chamber with a day length of 16 hr and a day/night temperature of 22/18°C. Humidity was maintained at 60% and the photosynthetic photon flux density (PPFD) at plant canopy height measured at 500 μmol/m^2^ s^−1^, giving a daily integrated photon flux of 28.8 mol/m^2^ day^−1^. Atmospheric CO_2_ in the chamber was the current ambient level. Plants were allocated to a drought and a control treatment, and all were first watered to field capacity every 3 days for 4–5 weeks. After this time, watering was completely stopped for individuals in the drought treatment. Controls continued to be watered every 3 days.

### 2.3 Shoot senescence and mortality

Depending upon germination success, up to 20 individuals per species were available to assess for senescence and death (Appendix 1). Six individuals per species were kept well‐watered as controls. Plants were visually assessed for shoot senescence and plant death every second day throughout the experiment. Senescence was counted as the days between transplanting and the full senescence of outer leaves and culms exposed to the external environment, that is, when there was no visible greenness on outer leaves and culms. The time until death was the number of days between transplanting and the point when there was no greenness visible on any of the leaves, culms, shoot apical meristems, or axillary buds. To ensure death had occurred, plants were then rewatered and observed for 2 weeks to check for regrowth. Any plants that showed regrowth were discarded from the mortality analyses. The difference in the time of occurrence between senescence and death was used as a measure of the time that any growing points were able to stay viable in a dormant state, from now on referred to as meristem survival.

### 2.4 Trait measurements

We measured stomatal conductance (g_s_), midday water potential, and predawn water potential on a subset of 8 species. For each of these species, one individual from the drought treatment and one control was measured every 3 days. Plants upon which measurements were to be taken were assigned a random sampling number to indicate the order in which they should be measured throughout the drought. A total of between six and ten individuals were measured per species over time, depending on how long it took for each species to close its stomata. An open gas exchange system (LI‐6400, LI‐COR Inc, Lincoln, Nebraska, USA) was used to measure stomatal conductance on one of the newest fully expanded leaves from each individual. The leaf was clipped into the chamber and allowed to equilibrate for less than 3 min, to gain a “snapshot” of physiological behavior under growth conditions. The chamber was set to a block temperature of 22 degrees, PPFD of 500 μmol/m^2^ s^−1^, flow rate of 300 μmol/s, and CO_2_ of 400 ppm to match the growth environment. Measures of stomatal conductance were always made between 5 and 7 hr after the lights in the chamber turned on. Where plants rolled their leaves under drought, leaves were unrolled before measurements were taken and leaf rolling recorded.

After making stomatal conductance measurements, midday water potential was measured and a black plastic bag placed over the plant to keep it in humid, dark conditions, allowing measure of predawn water potential (PDWP) to be taken the following morning. Leaf water potential is a measure of the resistance pathway for water movement and is also a function of soil water availability, evaporative demand, and soil conductivity. Predawn water potential is an indication of soil‐available water, as at this time the water potential of the leaf is in equilibrium with the soil. Water potentials were measured by removing one leaf, which was immediately placed in a Scholander pressure bomb (Model 600, PMS Instrument Company, Albany, Oregon, USA).

For each species, a threshold of g_s_ < 5 mmol/m^2^ s^−1^ was used to define stomatal closure and the predawn water potential at this threshold (Ψ_crit_) was recorded following (Craine et al., 2013). Midday water potential was plotted against predawn values, and a linear model fitted for each species, whose slope (*σ*) was used to indicate the relative sensitivity of plant hydraulic conductance to declining water availability (i.e., hydraulic vulnerability to water deficits (Martinez‐Vilalta et al., 2014). The intercept of this relationship (Λ) was taken as a simple measure of the maximum transpiration rate per unit of hydraulic transport capacity under well‐watered conditions (Martinez‐Vilalta et al., 2014).

Root measurements were taken on the six control plants for all 18 species. All of the growing medium was removed by washing the roots, and the area, diameter, and total length scanned using a root image analysis system (WinRHIZO, Regent Instruments, Quebec City, Canada). The roots were then dried at 70°C for 48 hr, the dry mass determined and used to calculate specific root length (SRL = length/dry mass). The above‐ground biomass of the control plants was also dried at 70°C for 48 hr and the dry mass determined.

### 2.5 Phylogenetic reconstruction

For each of the 18 species included in the study, sequences were obtained from Genbank for the chloroplast markers *trnLF, trnKmatK*, *ndhF,* and *rbcL* (see Appendix 1). Each marker was individually aligned using Muscle (Edgar 2004) and manual adjustments made. The four datasets were then concatenated, resulting in an alignment with 6,476 base pairs. The best‐fitting models of molecular evolution for each of marker were estimated using PartitionFinder (Lanfear et al., 2012). The *HKY + I* model for the site *rbcL*, GTR + G for *trnKmatK* and *ndhF* and HKY + G model for *trnLF* were applied to produce a time‐calibrated phylogenetic tree through Bayesian inference implemented in BEAST2 (Bouckaert et al., 2014). A log‐normal relaxed clock was used, with priors on divergence times modeled by a Yule process. A single run consisting of a single MCMC chain were run for 10,000,000 generations. Convergence of the runs was assessed in Tracer and the first 10% of the run discarded as burn‐in. All the trees sampled after burn‐in were pooled, and common ancestor node heights were plotted on a maximum clade credibility tree, which was used for comparative analyses (Appendix 2).

### 2.6 Statistical analyses

All statistical analysis was conducted using R 3.6.3 (R Core Team, 2013), using species means for each of the traits and environmental predictors.

We used a phylogenetic generalized least squares model (PGLS) implemented in the caper package in R (Orme, 2013) to assess the relationships between death, senescence, and meristem survival with each individual trait and MAP. PGLS accounts for phylogenetic autocorrelation in model residuals that is expected due to common ancestry.

## 3 RESULTS

### 3.1 Mortality

**FIGURE 1:**
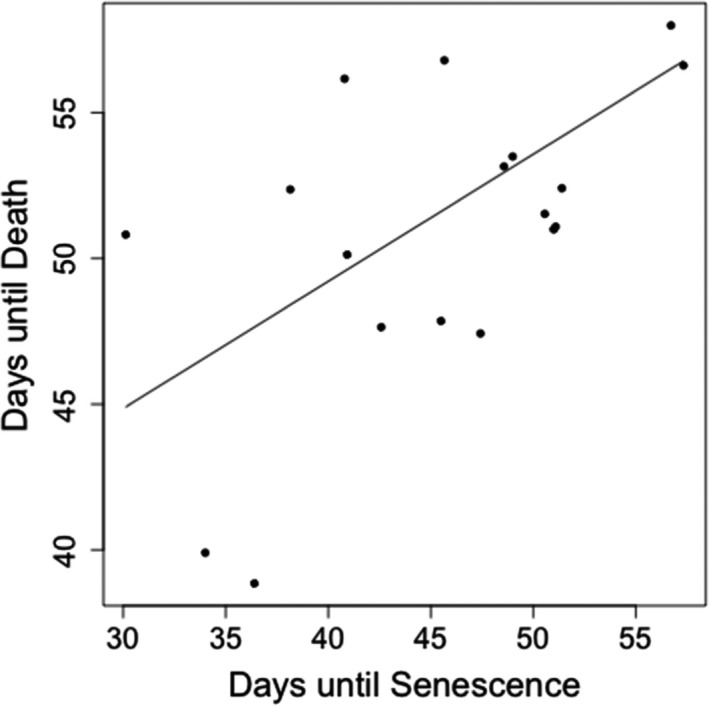
Relationship between the number of days until senescence and number of days until death (*r* ^2^ = 0.334, *λ* = 0.221, *p* = <.01)

At the end of the experiment, all the control plants that had been kept well‐watered were alive. We wanted to know whether interspecific variation in the number of days until death could be predicted by traits and climate, so performed a series of PGLS analyses on time until death, testing for relationships with each potential predictor (Table 1). A positive relationship was evident between death and senescence, with plants that stayed green the longest also surviving the longest under drought (*r* ^2^ = 0.334, *λ* = 0.275, *p *= <.01, slope = 0.417, *SE* = 0.135, *n* = 18) (Figure 1).

**TABLE 1:** The relationships between death, senescence, meristem survival, and the traits hypothesized to predict their variation

	Death				Senescence				Meristem Survival			
	*r* ^2^	*p* (*λ* = 1)	*λ*	*n*	*r* ^2^	*p* (*λ* = 1)	*λ*	*n*	*r* ^2^	*p* (*λ* = 1)	*λ*	*n*
MAP	0.023	ns	0	18	**0.203**	*	**0**	**18**	**0.490**	*	**0**	**10**
Root diameter	0.126	ns	0	18	**0.253**	*	**0**	18	−0.068	ns	0	10
*σ*	0.283	ns	0	12	−0.089	ns	0	12	0.275	ns	0	7
Λ	−0.099	ns	1	12	0.023	ns	1	12	0.044	ns	1	7
Ψ_crit_	0.211	ns	0	8	0.211	ns	0	8				
SRL	−0.034	ns	0	18	−0.057	ns	0	18	−0.107	ns	0	10
Senescence	**0.334**	**	0.275	**18**								

Note

Significant relationships are highlighted in bold, “*,” *p* < .05; “**,” *p* < .01; “***,” *p* < .001. *λ* is a measure of phylogenetic signal in the residuals of the model. A value of 1 indicates strong phylogenetic signal. A value of 0 indicates no phylogenetic signal.

In order to test for the possibility of plant size confounding our results, we tested the relationship between plant size with the number of days until death (adjusted *r* ^2^ = −0.051, *λ* = 0, *p *= n.s). and also plant size with senescence (adjusted *r* ^2^ = −0.018, *λ* = 0, *p* = n.s). We found no relationship between plant size and either of these variables (Appendices 3 and 4).

**FIGURE 2:**
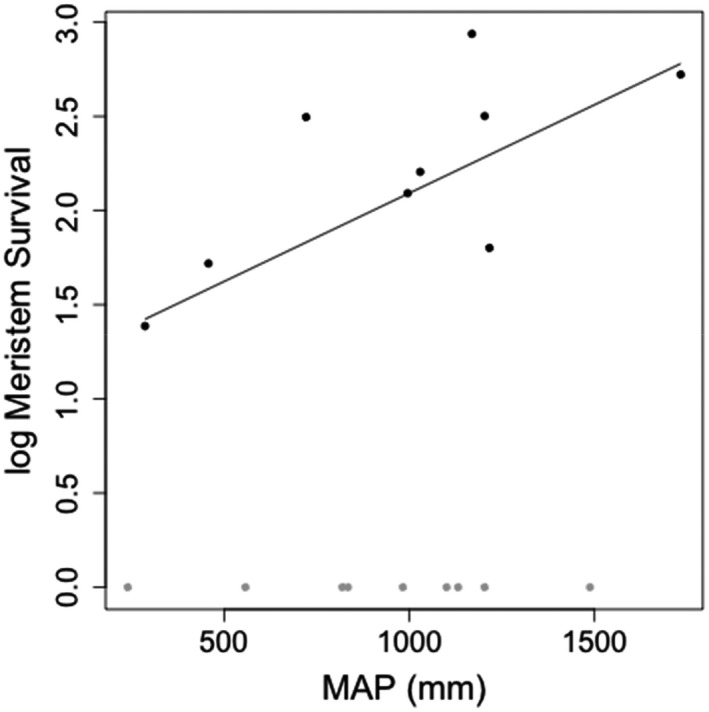
The relationship between meristem survival and mean annual precipitation (MAP). Meristem survival (days) was log‐transformed. Species with meristems that survived after the senescence of exterior aerial parts (i.e., drought tolerators) are shown in black. In contrast, species in which meristems did not survive any longer than exterior aerial parts (i.e., drought avoiders) are shown in gray. The line shows the relationship between meristem survival and MAP for drought tolerators (*r* ^2^ = 0.49, *λ* = 0, *p* = <.05)

Two strategies were identified in relation to greenness: species for which the senescence of external aerial parts corresponded to plant death; and species that were able to extend survival beyond leaf senescence via persistent meristems. Although death was not directly associated with variation in climatic variables across all the species in the experiment, if the analysis was confined to those species with persistent meristems, meristem survival after full senescence was strongly and significantly related to MAP (*r* ^2^ = 0.49, *λ* = 0, *p *= <.05, slope = 0.007, *SE* = 0.003, *n* = 9) (Figure 2). Species that exhibited the longest meristem survival after leaf senescence, meaning they have potential for resprouting for the longest time after full senescence, occupied environments with a high MAP.

### 3.2 Shoot senescence

**FIGURE 3:**
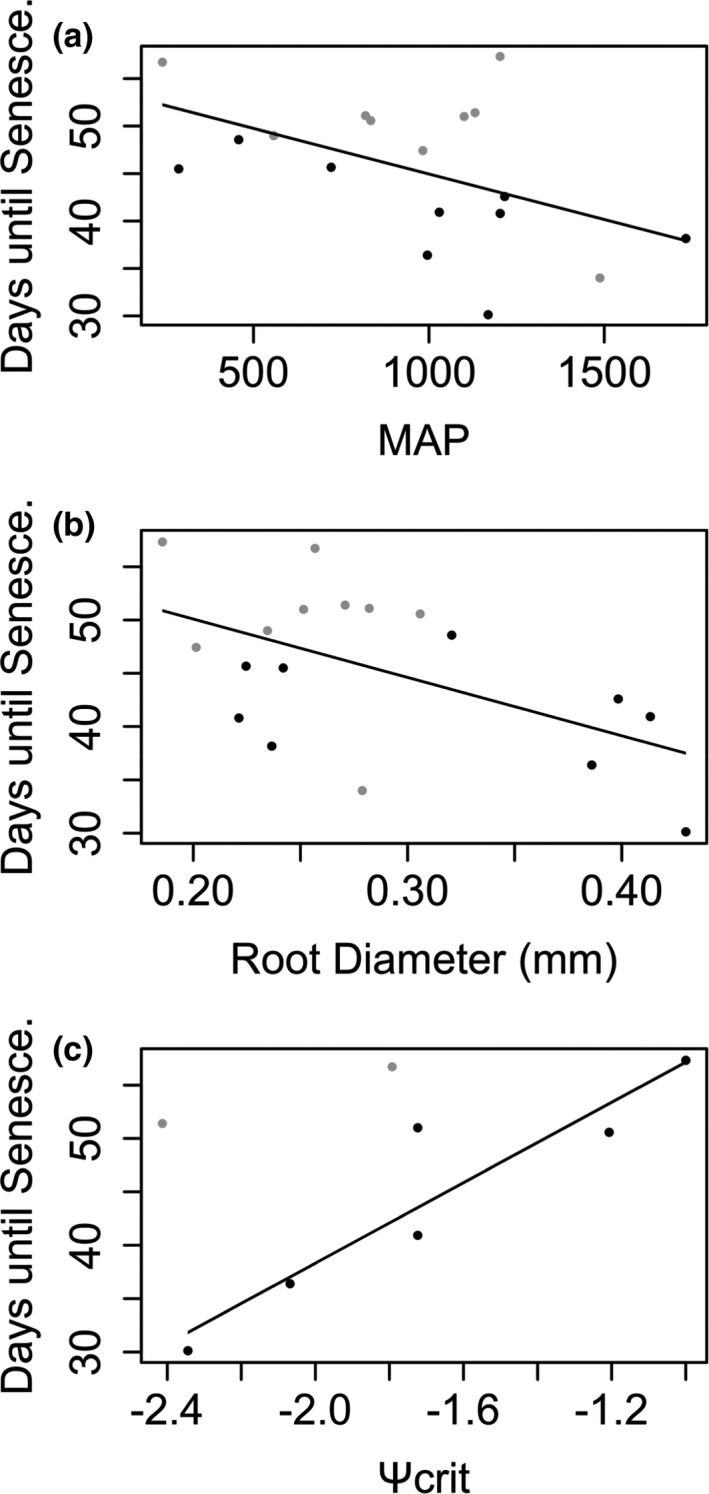
(a).The relationships between senescence and MAP (adjusted *r* ^2^ = 0.203, *λ* = 0, *p* = <.05). Species with meristems that were able to survive after senescence of exterior aerial parts (i.e., drought tolerators) are shown in black. Species without drought‐tolerant meristems (i.e., drought avoiders) are shown in gray (b). The relationship between time until senescence and root diameter (adjusted *r* ^2^ = 0.253, *λ* = 0, *p *= <.05. Species with meristems that were able to survive after senescence of exterior aerial parts (i.e., drought tolerators) are shown in black. Species without drought‐tolerant meristems (i.e., drought avoiders) are shown in gray (c).The relationship between Ψ_crit_ and days until senescence does not including leaf‐rolling species (*r* ^2^ = 0.827, *λ* = 0, *p *= <.01). In the bottom panel, these species are shown in black and species that roll leaves are shown in gray

**FIGURE 4:**
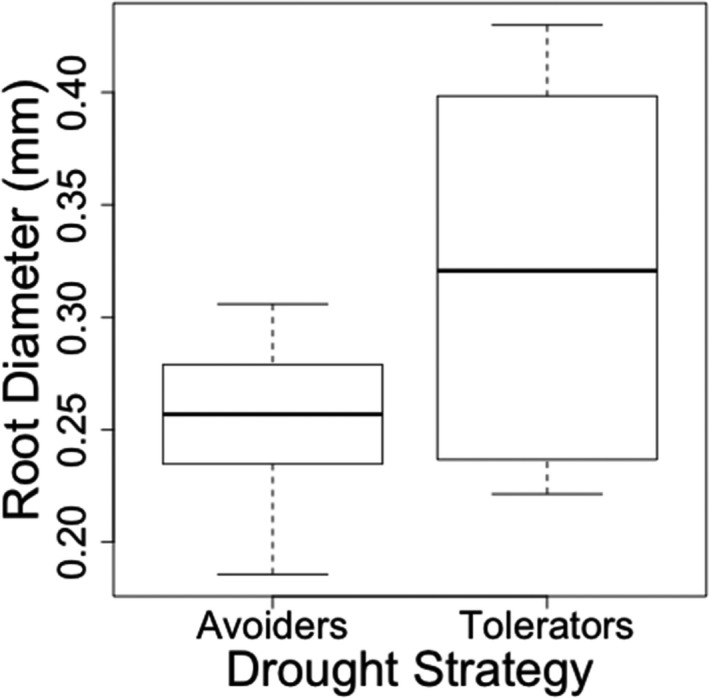
The difference in mean root diameter for drought tolerators (species with drought resistant meristems and the ability to resprout following full senescence) and drought avoiders (nonresprouting species). ANOVA showed significant differences between the two strategies (*F* = 4.32, *p* < .05)

Having established that senescence was a strong predictor of death, we then tested if variation in senescence could be explained by traits and climate. When looking at the relationship of senescence to individual predictors (Table 1), MAP was important in explaining interspecific variation in rates of senescence under drought (adjusted *r* ^2^ = 0.203, *λ* = 0, *p *= <.05, slope = −0.010, *SE* = 0.004, *n* = 18), as was root diameter (adjusted *r* ^2^ = 0.253, *λ* = 0, *p *= <.05, slope = −54.847, *SE* = 21.120, *n* = 18) (Figure 3a). Species that senesced quickly during the experimental drought treatment occupy wetter habitats and have larger root diameter (Figure 3b). We also found that species with the ability to resprout from meristems (drought tolerators) had significantly wider root diameter than species that stayed green throughout the drought (drought avoiders) (*F* = 4.32, *p* < .05) (Figure 4).

We found a positive but nonsignificant overall relationship between shoot senescence and Ψ_crit_, with species that senesced quickly always having a low Ψ_crit_, meaning that stomata stay open for longer with declining water potential (adjusted *r* ^2^ = 0.211, *λ* = 0, *p* = n.s, slope = 11.256, *SE* = 6.640, *n* = 8) (Figure 3c). Species that stayed greener for longer showed a wider range of stomatal responses. However, *Aeluropus lagopoides* and *Sporobolus indicus* were outliers to the overall pattern. These were the only species in the experiment to exhibit leaf rolling, and their leaves were unrolled to take the measurements of stomatal conductance. Leaf rolling, however, effectively reduces transpiration by decreasing the boundary layer conductance of the leaf, enabling stomata to stay open longer. When these two species were excluded from the analysis, the relationship became very strong (*r* ^2^ = 0.8269, *λ* = 0, *p *= <0.01, slope = 18.844, *SE* = 3.780, *n* = 6), with species that senesced quickly leaving stomata open for longer (Figure 3c).

### 3.3 Traits and climate

We found no significant relationships between individual traits and MAP (Appendix 5).

## 4 DISCUSSION

We evaluated whether mortality under drought could predict the position of species along a precipitation gradient and measured functional, physiological, and morphological traits that may explain variation in mortality. Species varied widely in the time until death under drought; however, mortality under drought was not related statistically to species distributions along global precipitation gradients. A similar pattern has also been observed in tropical forests whereby drought survival in itself is not correlated with MAP, because survival is primarily determined by deciduousness (Poorter & Markesteijn, 2008). We found that senescence of exterior parts was strongly related to MAP, with plants that stayed green for longer living in drier areas. There was a possibility that larger plants dried the pots fastest and would therefore be the quickest to either senesce or die. However, we are able to rule this out because there were no relationships between plant size and the number of days until death or the rate of senescence under drought.

We identified two strategies for survival. Some species maintained green shoots throughout the drought and the full senescence of exterior parts coincided with plant death. We refer to these species as “drought avoiders” because they avoid the adverse effects of water deficits by maintaining plant water potential as the soil dries. Drought avoiders use traits that increase access to water or delay water loss, for example narrow roots, which have a high surface area to volume which means that a larger volume of soil can be explored for the equivalent investment in root mass. Drought avoiders also exhibited early stomatal closure, to maintain plant water status and retain leaves throughout the drought (Levitt, 1980). An alternative strategy is adopted by “drought tolerators,” which senesce quickly, but where survival is extended via drought resistant meristems. These species do not maintain photosynthetic tissues throughout drought but are able to persist via meristems that can remain alive at low water potential.

The identification of these two strategies explains the absence of a relationship between death and MAP. Staying green for longer was the best strategy for survival as soil dried, and plants that stayed green for longer lived the longest. In habitats where water is limited, retaining green shoots as soil dries has the advantage that new leaves do not need to grow when rains occur, enabling photosynthesis to rapidly resume. In contrast, a drought tolerance strategy has the advantage of maintaining traits that are important for growth when rain is plentiful, for example high stomatal conductance. However, the physiological properties needed for rapid growth are incompatible with drought avoidance, and it seems likely that drought is therefore tolerated in an apparently dormant state throughout a long and predictable dry season.

Within the drought‐tolerating group of species, meristem survival under drought was associated with MAP. Species from the wettest habitats had the longest surviving meristems as soil dried, which is likely linked to the interaction between rainfall, seasonality, and fire. Grasslands in high precipitation environments are associated with a predictable dry season and frequent fires, whereas the dry season in arid grasslands is less predictable and fires are less frequent (Govender et al., 2006). The ability to resprout from meristematic tissue is an important trait in seasonally drought‐prone systems which are controlled by fire (Pausas et al., 2016; Simpson et al., 2020). We hypothesize that drought‐avoiding grass species will grow quickly under wet conditions, senesce rapidly under drought “creating fuel for grassland fires” but will have the ability to resprout rapidly from drought‐tolerant meristems following drought and fire.

We found that grass species with drought‐tolerant meristems have wider root diameter than drought avoiders. Resprouting shrubs in Mediterranean drought‐prone ecosystems have a wider root diameter (Paula & Pausas, 2011), where carbohydrates are stored to support regrowth after disturbance (Schutz et al., 2009). Differences in root structure between sprouting and nonresprouting shrubs reflect different foraging strategies, whereby nonresprouters more efficiently explore the upper soil layer via thinner, more branching roots (Paula & Pausas, 2011). However, the root structure of resprouting species enabled carbon storage and deep soil penetration. It is likely that an ability to resprout in grassland species is facilitated by wide roots and stored underground carbon reserves. Future work should investigate the relationship between growth rate, storage of nonstructural carbohydrates and resprouting.

A trend for decreasing root diameter with lower rates of canopy senescence was observed across all species (including both drought tolerators and drought avoiders). The ability to effectively take up water via roots is an important part of drought tolerance (Rieger & Litvin, 1999) and also in determining maximum rates of gas exchange (Brodribb & Feild, 2000). Water transport in roots of small diameter is more efficient than in wider roots. This is because soil‐root hydraulic conductivity is increased by having a larger surface area in contact with the soil, (Rieger & Litvin, 1999) and is expected to increase water acquisition as soil dries (Wasson et al., 2012).

Of the other traits measured, although the sample size was small, our results indicate that stomatal regulation is important in remaining green for longer, but only after species that rolled leaves were removed from the analysis. The rolling of leaves reduces boundary layer conductance and enables stomatal conductance on the rolled leaf surface to remain higher for longer as the drought progresses (Taylor et al., 2014). This is because leaf rolling increases the boundary layer resistance near the leaf surface to create a humid microclimate (Kadioglu & Terzi, 2007). By rolling leaves, plants are able to remain photosynthetically active under drought, reducing the risk of carbon starvation, while limiting water loss (Knapp, 1985).

We found no direct relationship between Ψ_crit_, root diameter, nor any other traits with MAP. MAP may be too coarse a measurement of plant available water. Future work could consider other environmental gradients linked to MAP, for example the temperature and/or vapor pressure deficit values along a precipitation gradient, which are known to influence functional trait composition in forests (Wieczynski et al., 2019). Other traits may also be important in drought performance, but were beyond the scope of this study. For example, osmotic adjustment helps to maintain cell turgor and therefore sustain physiological processes, including stomatal opening and photosynthesis under drought (Bartlett et al., 2012; Blum et al., 1983; Ludlow & Muchow, 1990). A great diversity of covarying traits coexist in dry habitats (Hernandez et al., 2010), and different combinations of these traits may act to achieve the same effect.

In conclusion, this study shows that survival under drought could not explain species distributions along precipitation gradients because this was predominantly associated with senescence and meristem persistence. Species whose leaf canopies stayed green for longer were associated with arid environments, whereas species that senesced quickly but persisted for longer without leaves grew in wet regions. Based on these findings, two strategies were identified in response to declining soil water: (a) drought avoiders that retained green photosynthetic shoots throughout drought; and (b) drought tolerators with quickly senescing shoots but with the ability to extend survival via drought resistant meristems. Staying green for longer resulted in the longest survival, and traits that facilitated this were small root diameter and either leaf rolling or isohydric stomatal regulation. Plants that senesced quickly had wider root diameter and anisohydric stomatal regulation. Our results suggest that the global distributions of C_4_ grasses can be predicted by variation in rates of senescence and meristem survival, root traits, and stomatal strategy. The work shows the value of these traits for understanding the distributions of herbaceous species in relation to moisture availability.

## CONFLICT OF INTEREST

None declared.

## AUTHOR CONTRIBUTIONS

**Emma C. Jardine:** Conceptualization (equal); data curation (equal); formal analysis (lead); methodology (equal); visualization (equal); writing–original draft (lead); writing–review and editing (lead). **Gavin H. Thomas:** Conceptualization (supporting); formal analysis (supporting); methodology (supporting); writing–review and editing (supporting). **Colin P. Osborne:** Conceptualization (equal); formal analysis (supporting); funding acquisition (lead); methodology (supporting); writing–review and editing (supporting).

## Data Availability

All data are publicly available https://doi.org/10.5061/dryad.2ngf1vhms

## APPENDIX 1

The number of individuals that were included in the analyses (excluding the 6 control plants), the number of geo‐referenced records from GBIF after cleaning of the data that were used to calculate mean annual precipitation and precipitation seasonality for each species and the Genbank accession numbers for sequences used to build the phylogenetic tree used in analyses

**Table:**

Species	Accession	Source	MAP (mm)	No. Individuals	No. Geo‐referenced records	Genbank Accession Numbers		matK	trnL
						*rbcL*	*ndhF*		
*Aeluropus lagopoides* (L.) Thw.	PI 439902	USDA	239.912	20	34		GU359591.1		GU360013.1
*Astrebla lappacea* (Lindl.) Domin	PI 284733	USDA	1,100.5	9	306	JN681651.1	JN681599.1	AF144589.1	GU360009.1
*Calamovilfa longifolia* (Hook.) Scribn.	PI 477995	USDA	457.67	19	10	EF125104.1	GU359716.1		EF137559.1
*Chloris gayana* Kunth	12779	AusTRCF	995.49	20	419	AM849409.1	AM849205.1	AF164424.1	KR738428.1
*Cymbopogon flexuosus* (Nees) W. Watson	not known	Supplier: B&T World Seeds	1732.41	19	16	KP087913.1	AF117404.1	KT309064.1	DQ004971.1
*Digitaria eriantha* Steud.	319760	AusTRCF	819.2	20	303	HE573375.1	HE573497.1	HE574068.1	KP057660.1
*Echinochloa pyramidalis* (Lam.) Hitchc. & Chase	PI 299897	USDA	1,216.13	17	269	KR737524.1		KR735143.1	KR738671.1
*Eleusine indica* (L.) Gaertn.	PI 217609	USDA	1,168.29	17	345	EF125108.1	AM849151.1	AF144580.1	EF156691.1
*Eragrostis lugens* Nees	PI 203862	USDA	982.62	20	198		GU359704.1		GU990387.1
*Eulalia aurea* (Bory) Kunth	PI 371930	USDA	557.51	6	2,812	AM849410.1	AM849213.1	HE574011.1	
*Panicum lanipes* Mez	Unknown	Unknown	286.51	7	58				AY142732.1
*Paspalum scrobiculatum* L.	PI 435944	USDA	1,487.64	10	565	LN907994.1	LN908157.1	LN906759.1	AB817562.1
*Schizachyrium sanguineum* (Retz.) Alston	Unknown	Supplier: Silverhill seeds	1,203.38	12	273	LN908005.1	LN908168.1	LN906770.1	DQ004993.1
*Sorghastrum nutans* (L.) Nash	322519	AusTRCF	1,029.45	15	318	FR821342.1	FR821360.1	FR821326.1	DQ005001.1
*Sporobolus indicus* (L.) R. Br.	PI 310427	USDA	1,131.52	17	332	HE575834.1	HE575785.1	HE575870.1	EF156732.1
*Stenotaphrum secundatum* (Walt.) Kuntze	PI 600734	USDA	1,203.15	16	369	EF125139.1	AY029684.1	KC123431.1	EU939985.1
*Themeda triandra* Forsk.	90630	Kew	721.24	15	434	LN908022.1	LN908185.1	LN906787.1	DQ005005.1
*Urochloa mosambicensis* (Hack.) Dandy	319622	AusTRCF	834.3	15	370		FJ486516.1		GU594532.1

## APPENDIX 2

The phylogenetic tree showing relationships between the taxa included in this study produced by Baysian inference

## APPENDIX 3

The relationship between above‐ground biomass and number of days until senescence under drought (adjusted *r* ^2^ = −0.018, *λ* = 0, *p* = n.s)

## APPENDIX 4

The relationship between above‐ground biomass and number of days until death under drought (adjusted *r* ^2^ = −0.051, *λ* = 0, *p* = n.s)

## APPENDIX 5

The relationship between traits and mean annual precipitation

**Table:**

	*R* ^2^	*p*	*λ*
Root diameter	−0.0534	ns	0
*σ*	−0.0602	ns	0
SRL	0.1159	ns	0
Λ	0.0738	ns	0
Ψcrit	−0.2487	ns	0
